# Patients with Tuberculosis Disease Have *Mycobacterium tuberculosis*-Specific CD8 T Cells with a Pro-Apoptotic Phenotype and Impaired Proliferative Capacity, Which Is Not Restored following Treatment

**DOI:** 10.1371/journal.pone.0094949

**Published:** 2014-04-16

**Authors:** Cheryl L. Day, Noella D. Moshi, Deborah A. Abrahams, Michele van Rooyen, Terrence O'rie, Marwou de Kock, Willem A. Hanekom

**Affiliations:** 1 South African Tuberculosis Vaccine Initiative (SATVI) and School of Child and Adolescent Health, Institute of Infectious Diseases and Molecular Medicine, University of Cape Town, Observatory, South Africa; 2 Department of Microbiology and Immunology, Emory University School of Medicine, Atlanta, Georgia, United States of America; 3 Department of Global Health, Rollins School of Public Health, Emory University, Atlanta, Georgia, United States of America; 4 Emory Vaccine Center, Emory University, Atlanta, Georgia, United States of America; University of Palermo, Italy

## Abstract

CD8 T cells play a critical role in control of chronic viral infections; however, the role of these cells in containing persistent bacterial infections, such as those caused by *Mycobacterium tuberculosis* (Mtb), is less clear. We assessed the phenotype and functional capacity of CD8 T cells specific for the immunodominant Mtb antigens CFP-10 and ESAT-6, in patients with pulmonary tuberculosis (TB) disease, before and after treatment, and in healthy persons with latent Mtb infection (LTBI). In patients with TB disease, CFP-10/ESAT-6-specific IFN-γ^+^ CD8 T cells had an activated, pro-apoptotic phenotype, with lower Bcl-2 and CD127 expression, and higher Ki67, CD57, and CD95 expression, than in LTBI. When CFP-10/ESAT-6-specific IFN-γ^+^ CD8 T cells were detectable, expression of distinct combinations of these markers was highly sensitive and specific for differentiating TB disease from LTBI. Successful treatment of disease resulted in changes of these markers, but not in restoration of CFP-10/ESAT-6-specific CD8 or CD4 memory T cell proliferative capacity. These data suggest that high mycobacterial load in active TB disease is associated with activated, short-lived CFP-10/ESAT-6-specific CD8 T cells with impaired functional capacity that is not restored following treatment. By contrast, LTBI is associated with preservation of long-lived CFP-10/ESAT-6-specific memory CD8 T cells that maintain high Bcl-2 expression and which may readily proliferate.

## Introduction

The vast majority of individuals infected with *Mycobacterium tuberculosis* (Mtb) never develop clinical symptoms of tuberculosis (TB) disease [1], thus providing compelling evidence of the ability of the host immune response to successfully control the pathogen. However, host mechanisms of protection against development of TB disease have not been fully elucidated.

A clear role for CD4 T cells in immunological control of Mtb infection has been demonstrated in mouse models: increased susceptibility to Mtb infection was shown in MHC class II-/- mice [2], and rapid reactivation of Mtb replication and decreased survival followed experimental antibody-mediated depletion of CD4 T cells [3]-[5]. However, a direct role for Mtb-specific CD8 T cells in mediating protective immunity to Mtb is less clear. Similar to CD4 T cells, CD8 T cells can produce IFN-γ and TNF-α, which directly activate macrophages and promote the production of nitrogen and oxygen radicals to suppress Mtb growth [6]-[8]. CD8 T cells can kill Mtb-infected cells by production of cytotoxic molecules such as perforin and granulysin, and/or may modulate the microenvironment by production of cytokines [9]-[11]. CD8 T cells have been shown to mediate suppression of Mtb growth in vitro in blood-derived macrophages [12] as well as autologous alveolar macrophages [13]. Mtb infection of CD8 T cell-deficient mice, or depletion of CD8 T cells in vivo following infection, has been reported to result in higher bacterial burdens [14]-[16]. However, other similar murine studies have reported no differences in bacterial load or survival in the absence of CD8 T cells [4], [5], [17]. In rhesus macaques, depletion of CD8 T cells results in loss of Bacille Calmette Guérin (BCG) vaccine-induced immunity and reduced immunity to Mtb [18].

MHC class I-restricted CD8 T cells recognizing a variety of Mtb antigens have been detected in individuals with latent Mtb infection (LTBI) and with active TB disease [19]-[29]. IFN-γ-producing CD8 T cells specific for epitopes within the immunodominant antigens CFP-10 and ESAT-6 have been more readily detectable in peripheral blood of patients with active TB disease, compared with individuals with LTBI [30]-[32]. Despite higher frequencies of cytokine-producing Mtb-specific CD8 T cells in peripheral blood of patients with TB, other functions of Mtb-specific CD8 T cells, including cytolytic activity [33], production of cytotoxic molecules [34], and proliferation [30], [32], [35], have been reported to be impaired in patients with active TB. Immunoregulatory molecules that have been associated with CD8 T cell dysfunction and immune exhaustion in chronic viral infections, including CD160, programmed death receptor 1 (PD-1), and 2B4 [36], have been reported to be expressed at low levels on CFP-10 and ESAT-6-specific CD8 T cells, both in the setting of latent infection and active TB disease [32]. Moreover, expression of the immunoregulatory molecule TIM-3, which has also been associated with T cell dysfunction in chronic viral infections [37], has been reported to be associated with high effector function capacity when expressed by Mtb-specific CD4 and CD8 T cells [38]. Thus phenotypic molecules that are associated with functional impairment of antigen-specific CD8 T cells in the setting of active TB disease in humans have not been well defined.

Progressive dysfunction of antigen-specific CD8 T cells has been described in the context of chronic viral infections [36], [39]–[42]. This dysfunction correlates with antigen load and has been associated with distinct phenotypic profiles of antigen-specific T cells, such as loss of expression of CD127, the IL-7 receptor α chain [43]–[45], and increased expression of inhibitory receptors [46], [47]. Antigen-specific CD8 T cells in chronic infection also express low levels of the anti-apoptotic molecule Bcl-2 [48]–[50] and high levels of pro-apoptotic CD95 (Fas), rendering these cells more susceptible to apoptosis and deletion [51]–[53]. Upregulation of CD57 expression by CD8 T cells in chronic infections has been linked to replicative senescence and greater propensity to antigen-induced apoptosis [54]–[56].

The relationship between mycobacterial antigen load and the phenotypic profiles of CFP-10/ESAT-6-specific CD8 T cells in humans has not been well described. We hypothesized that functional impairment of CFP-10/ESAT-6-specific CD8 T cells in patients with active TB disease and high mycobacterial load would be associated with dysregulated expression of phenotypic molecules associated with memory, activation, senescence, and apoptosis. We therefore examined the phenotypic profiles and proliferative capacities of CFP-10/ESAT-6-specific CD8 T cells in healthy asymptomatic individuals with LTBI and in patients with active pulmonary TB disease, before and after successful completion of anti-TB treatment.

## Materials and Methods

### Ethics Statement

All participants provided written informed consent to participate in the study, which was approved by the Human Research Ethics Committee of the University of Cape Town, and by the Western Cape Department of Health.

### Study population and sample collection

HIV-uninfected persons between 18–65 years of age with latent Mtb infection (LTBI) or with pulmonary tuberculosis disease (TB) were recruited from the Cape Town region of South Africa. Persons with LTBI were identified as healthy, asymptomatic individuals (defined as individuals with negative symptom screening for all of the following TB-associated symptoms: persistent cough, fever, night sweats, weight loss, or hemoptysis), with IFN-γ^+^ CD4 or CD8 T cells to CFP-10 and/or ESAT-6 peptide pools in a whole blood intracelluar cytokine staining assay [30], and with no previous history of TB diagnosis or treatment. All patients with pulmonary TB disease had either a positive sputum smear microscopy, a positive sputum culture for Mtb, or both. In those with TB disease, peripheral blood was collected between 0 and 7 days of starting standard drug treatment, which consisted of 2 months of isoniazid, rifampicin, pyrazinamide, and ethambutol, followed by 4 months of isoniazid and rifampicin. TB treatment was provided according to South African national health guidelines, which include provisions for directly observed treatment (DOT) for monitoring of adherence. Drug suscepbility testing was conducted in accordance with the South African National Tuberculosis Control Programme guidelines; all patients tested for drug susceptibility were sensitive to first line TB drugs. Some patients with TB disease were followed longitudinally, with additional blood samples obtained at 2, 6, and 12 months after starting treatment. All TB patients followed longitudinally on treatment had an excellent response to treatment, as indicated by resolution of symptoms and two negative sputum smears, taken on two separate occasions, by the end of the 6 month treatment period.

### Antigens

15-mer peptides overlapping by 10 amino acids were synthesized to correspond with the sequence of ESAT-6 and CFP-10. A pool of overlapping peptides spanning the pp65 protein of human cytomegalovirus (HCMV) was included in the intracellular cytokine staining phenotyping assays (see below) for analysis of T cell phenotypes specific for a chronic antigen other than Mtb. The HCMV pp65 Peptide Set was obtained through the NIH AIDS Reagent Program, Division of AIDS, NIAID, NIH. Peptide pools for each of these were used at a concentration of 1.25 µg/ml/peptide in PBMC ICS assays (see below), and at 0.1 µg/ml/peptide in proliferation assays (see below). Staphylococcal enterotoxin B (SEB; Sigma-Aldrich) was used at 1 µg/ml in the ICS assays, and at 0.1 µg/ml in proliferation assays.

### Intracellular cytokine staining (ICS) phenotyping assay

Cryopreserved PBMCs were thawed in medium containing deoxyribonuclease I (DNAse; 100 µg/ml; Sigma-Aldrich), washed, and rested for at least 6 hours at 37°C. Peptide pools were then added for a total of 7 hours, with Brefeldin A (10 µg/ml) added after the first 2 hours. Cells were stimulated with SEB or incubated with no antigen, as positive and negative controls, respectively. Cells were then washed in PBS and stained with Vivid, fixed with FACS Lysing Solution, and permeabilized with Perm/Wash Buffer (BD Biosciences). Cells were stained with following mAbs: anti-CD3 allophycocyanin-H7 (SK7), anti-IFN-γ Alexa Fluor 700 (B27), anti-Ki67 PE (B56), anti-CD127 PerCP-Cy5.5 (HIL-7R-M21), all from BD Biosciences, anti-CD8 QDot605 (3B5) from Life Technologies, anti-CD57 APC (NK-1), and anti-CD95 PE-Cy7 (DX2), both from BioLegend. After 1 hour, cells were washed in Perm/Wash Buffer and acquired on a BD LSRII flow cytometer.

### PBMC proliferation assay

A six-day proliferation assay was performed as previously described [30]. Briefly, freshly isolated peripheral blood mononuclear cells (PBMCs) were labeled with CellTrace Oregon Green 488 carboxylic acid diacetate, succinimidyl ester (OG; Life Technologies). OG-labeled cells were incubated for 6 days with either Mtb peptide pools, or the positive control SEB, or no antigen (negative control). Cells were then stained with LIVE/DEAD Fixable Violet Dead Cell Stain (Vivid; Life Technologies), fixed with FACS Lysing Solution (BD Biosciences), and stained with anti-CD4 Qdot605 (S3.5, Life Technologies), anti-CD3 allophycocyanin-H7 (SK7), and anti-CD8 PerCP-Cy5.5 (SK-1), both from BD Biosciences.

### Flow cytometry

All flow cytometry data were acquired using a BD LSRII flow cytometer with FACSDiva software (v6.0; BD Biosciences), and analyzed using FlowJo (v9.5.2; TreeStar). Lymphocytes were gated by morphological parameters (forward scatter and side scatter). T cells were identified as CD3^+^ lymphocytes; live cells were identified as Vivid^lo^ cells; proliferating cells were identified as OG^lo^ cells. In ICS phenotyping assays, CFP-10/ESAT-6-specific CD8 T cells were defined as Vivid^l^°CD3^+^CD8^+^IFN-γ^+^ T cells following stimulation with CFP-10 or ESAT-6 peptide pools. As confirmed in a separate PBMC ICS assay, >90% of CD3^+^CD8^−^IFN-γ^+^ T cells following stimulation with CFP-10 or ESAT-6 peptide pools were found to be CD4^+^ (Fig. S3), and are thus referred to as CFP-10/ESAT-6-specific CD4 T cells. Co-expression patterns of phenotypic markers were analyzed using Pestle v1.7 (Mario Roederer, Vaccine Research Center, National Institute of Allergy and Infectious Diseases, National Institutes of Health) and Spice v5.22 [57].

### Data analysis

Responses in the ICS and proliferation assays were considered positive if the frequency of cytokine-producing or proliferating T cells in the stimulated sample was greater than the median plus 3 times the median absolute deviation of the negative control samples. In addition, a minimum of 20 IFN-γ^+^ T cell events above background cytokine production was required for subsequent phenotypic analysis in the ICS assay. Statistical analyses were performed using Prism v6.0c (GraphPad Software, Inc.). Differences between groups were assessed using the Mann-Whitney *U* test. The Wilcoxon matched pairs test was used to assess differences in CFP-10/ESAT-6-specific T cell responses within the same donor at different time points. To evaluate the predictive value of CFP-10/ESAT-6-specific CD8 T cell phenotypic profiles, a receiver operator characteristic (ROC) curve was generated and logistic regression analysis performed. The predictive value of each phenotypic pattern was evaluated by analysis of the area under the curve (AUC). P values in phenotypic analyses were corrected for multiple comparisons using Bonferroni correction to control the family-wise error rate. For comparison of three or more groups, a non-parametric Kruskal-Wallis test was performed, followed by the Dunn's post-test to account for multiple comparisons.

## Results

### Participants

Two groups of adults from the Cape Town region of South Africa were recruited for this study: healthy, asymptomatic adults with LTBI, and patients with active pulmonary TB disease. All participants were seronegative for HIV-1 antibodies. Characteristics of the study participants are summarized in Table 1.

**Table 1:** Characteristics of study population.

Participant Group	*n*	AFB Sputum Smear Positive, n (%)	TB Tx, Days (Range)^b^	Age, Years (Range)^c^	Sex (% Male)
LTBI	42	N/A	N/A	25 (18–50)	40%
TB	47	39 (83%)^a^	4 (0–7)	38 (20–65)^d^	70%^d^

a The remaining 8 individuals with TB were sputum smear-negative, culture positive for *M. tuberculosis*.

b Values denote median number of days on anti-TB treatment at first sample collection (range).

c Values denote median (range).

d *p*<0.05, compared with LTBI.

N/A, not applicable.

### CFP-10/ESAT-6-specific CD8 T cells have a short-lived, pro-apoptotic phenotype in TB disease patients, compared with persons with LTBI

Previous studies of chronic viral infections have clearly demonstrated dysfunction of virus-specific CD8 T cells in the context of persistent antigen stimulation, as well as distinct phenotypic profiles when chronic and acute infections were compared [36]. We have previously found differential functional capacities of peripheral blood CFP-10/ESAT-6-specific CD8 T cells in humans: those with active disease were more likely to have cells producing cytokines ex vivo, whereas those with LTBI characterstically had cells with robust proliferative capacity [30].

**Figure 1:**
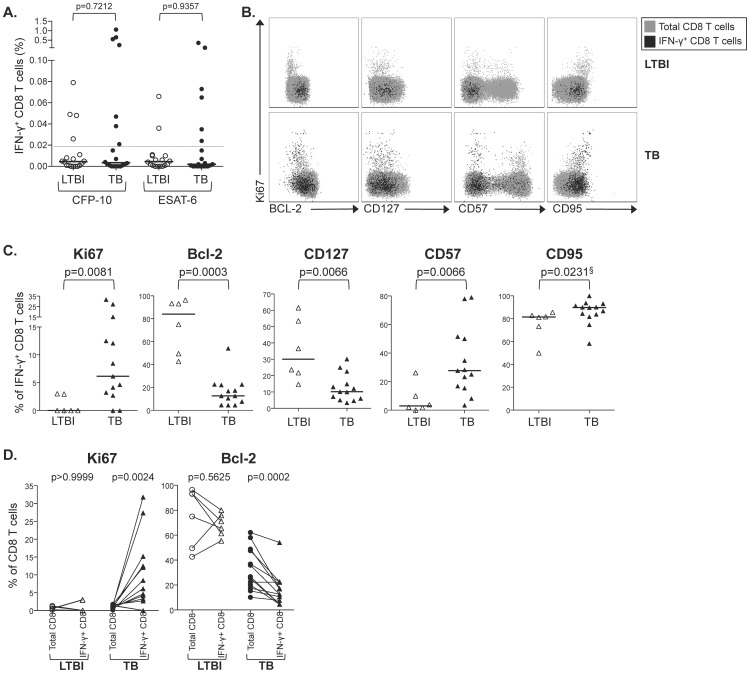
CFP-10/ESAT-6-specific CD8 T cells in patients with TB have a short-lived, pro-apoptotic phenotype. PBMCs from individuals with LTBI (n = 18) and patients with TB disease (n = 20) were stimulated for 6 hours with CFP-10 and ESAT-6 peptide pools; intracellular IFN-γ production was measured in CD8 T cells by flow cytometry. (**A**) Frequencies of CFP-10 and ESAT-6-specific IFN-γ^+^ CD8 T cells in persons with LTBI and patients with TB disease. Horizontal lines represent the median. Data are shown after subtraction of background cytokine production in the negative control condition. The dotted line separates individuals who met the criteria for a positive CD8 T cell response for further phenotypic analyses (above dotted line: n = 6 LTBI; n = 13 TB disease), and individuals in whom the frequency of IFN-γ^+^ CD8 T cells was too low to meet the criteria for further phenotypic analyses (below dotted line). (**B**) Representative flow cytometry data from an individual with LTBI and a patient with TB disease, following stimulation of PBMCs with an ESAT-6 peptide pool. Grey cells indicate the total CD8 T cell population (gated on VIVID^l^°CD3^+^CD8^+^ cells); black cells indicate ESAT-6-specific CD8 T cells (gated on VIVID^l^°CD3^+^CD8^+^IFN-γ^+^ cells). (**C**) Summary data of the percentage of specific IFN-γ^+^ CD8 T cells expressing Ki67, Bcl-2, CD127, CD57 and CD95 in individuals with LTBI and TB disease. Differences were assessed using the Mann-Whitney test. § indicates p values that did not remain significant after applying the Bonferroni correction for multiple comparisons. (**D**) Comparison of the expression of intracellular Ki67 and Bcl-2 between the total CD8 T cell population and specific IFN-γ^+^ CD8 T cells within the same individual. Only individuals meeting the criteria for a positive CD8 T cell response in the ICS assay were included in this paired analysis (n = 6 LTBI; n = 13 TB disease). Differences were assessed using the Wilcoxon matched-pairs test.

We hypothesized that the differential functional capacites of CFP-10/ESAT-6-specific CD8 T cells observed in individuals with LTBI and patients with TB disease would associate with expression of cellular markers of activation, senescence, and susceptibility to apoptosis. Thus, we measured expression of Ki67, Bcl-2, CD127, CD57, and CD95 among CFP-10/ESAT-6-specific CD8 T cells, identified by intracellular IFN-γ expression after short-term stimultion of PBMCs with CFP-10 and ESAT-6 peptide pools (Figure 1 and Figure S1). Of 38 individuals analyzed in our ICS phenotyping assay (n = 18 LTBI and n = 20 TB disesase), 6 persons with LTBI (33%) and 13 patients with TB disease (65%) had positive CFP-10 or ESAT-6-specific CD8 T cell responses that were subjected to further phenotypic analyses (Figure 1A, B). Compared with persons with LTBI, CFP-10/ESAT-6-specific CD8 T cells from TB diseased patients had significantly higher expression of Ki67, CD57, and CD95, and lower expression of the anti-apoptotic molecule Bcl-2 and memory T cell marker CD127 (Figure 1C).

We next directly compared expression of these molecules between the antigen-specific and total CD8 T cell populations within the same individual. Compared with total CD8 T cells, Ki67 expression was upregulated and Bcl-2 expression was downregulated by CFP-10/ESAT-6-specific CD8 T cells from patients with TB disease. By contrast, in individuals with LTBI, expression of Ki67 and Bcl-2 was similar between CFP-10/ESAT-6-specific CD8 T cells and the total CD8 T cell population (Figure 1D). Together these data indicate that modulation of expression of Ki67 and Bcl-2 within CFP-10/ESAT-6-specific CD8 T cells is unique to patients with active disease, and provide further evidence of an activated, pro-apoptotic population of CFP-10/ESAT-6-specific CD8 T cells in the context of high bacterial load.

### Co-expression patterns of Bcl-2, CD57, and CD95 by CFP-10/ESAT-6-specific CD8 T cells distinguish individuals with LTBI and patients with TB disease

**Figure 2:**
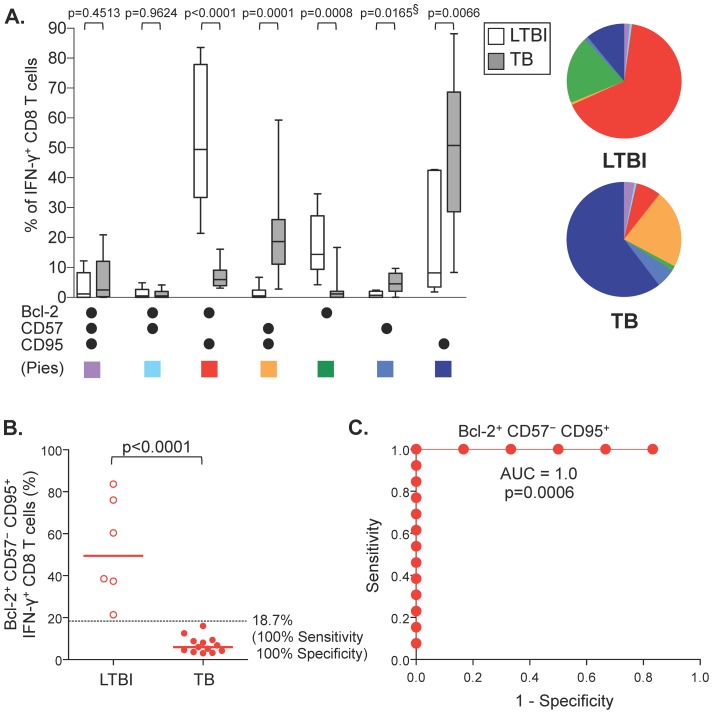
Differential co-expression patterns of Bcl-2, CD57 and CD95 by CFP-10/ESAT-6-specific CD8 T cells distinguish individuals with LTBI and patients with TB. Boolean analysis was performed to determine co-expression patterns of Bcl-2, CD57, and CD95 by CFP-10 and ESAT-6-specific CD8 T cells detected by ICS, as described in Figure 1. (A) Co-expression patterns of Bcl-2, CD57, and CD95 by specific IFN-γ^+^ CD8 T cells detectable in individuals with LTBI (open bars; n = 6) and patients with TB disease (grey bars; n = 13). Cells were gated on VIVID^l^°CD3^+^CD8^+^IFN-γ^+^ cells. Differences were assessed using the Mann-Whitney test. § indicates p values that did not retain statistical significance after applying the Bonferroni correction for multiple comparisons. Pie graphs indicate the median proportion of each population contributing to the total specific IFN-γ^+^ CD8 T cell response. (B) Comparison of the proportion of Bcl-2^+^CD57^−^CD95^+^ cells contributing to the total CFP-10 and ESAT-6-specific CD8 T cell responses in individuals with LTBI and patients with TB disease. Horizontal lines represent the median. Differences were assessed using the Mann-Whitney test. The dotted line indicates the cut-off (18.7%) that distinguishes individuals with LTBI and TB disease, with 100% specificity and 100% sensitivity. (C) Receiver operator characteristic (ROC) curve indicating the sensitivity and specificity of the proportion of CFP-10/ESAT-6-specific CD8 T cells that are Bcl-2^+^CD57^−^CD95^+^ to distinguish individuals with LTBI and TB disease. An area under the ROC curve (AUC) analysis was performed to further evaluate the performance of this phenotypic expression profile in distinguishing individuals with LTBI and TB disease.

We next used Boolean analysis to analyze co-expression patterns of Bcl-2, Ki67, CD127, CD57, and CD95 to determine if phenotypically distinct populations of CFP-10/ESAT-6-specific CD8 T cells differed significantly between individuals with LTBI and active disease. A combination of Bcl-2, CD57, and CD95 expression allowed differentiation of individuals with LTBI from patients with TB disease (Figure 2A and data not shown). The majority of CFP-10/ESAT-6-specific CD8 T cells in persons with LTBI co-expressed Bcl-2 and CD95, in the absence of CD57; by contrast, the majority of CFP-10/ESAT-6-specific CD8 T cells in patients with TB disease expressed CD95, with or without CD57 co-expression, but lacked Bcl-2 expression (Figure 2A). Cells expressing Ki67, a nuclear protein indicative of cell turnover in vivo, were found almost exclusively within the Bcl-2^−^CD57^−^CD95^+^ subset (data not shown). The proportion of CFP-10/ESAT-6-specific CD8 T cells displaying a Bcl-2^+^CD57^−^CD95^+^ phenotype demonstrated the greatest power to distinguish LTBI from TB disease (Figure 2B), with 100% sensitivity and specificity (Figure 2C). In addition, the proportion of Bcl-2^−^CD57^+^CD95^+^ and of Bcl-2^+^CD57^−^CD95^−^ CFP-10/ESAT-6-specific CD8 T cells classified individuals with LTBI and TB disease with >90% sensitivity and specificity (Figure S2).

### Pro-apoptotic phenotype of CFP-10/ESAT-6-specific CD4 T cells in patients with TB disease

**Figure 3:**
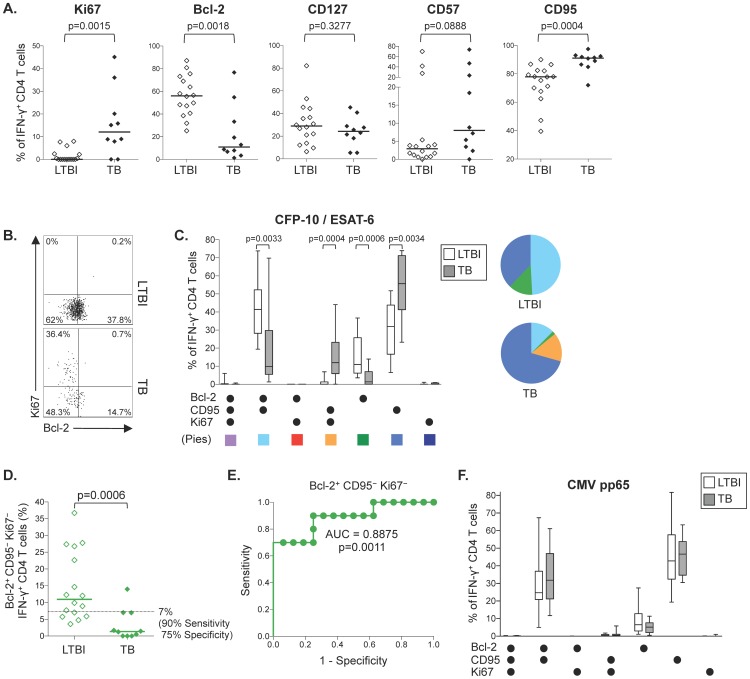
Pro-apoptotic phenotype of CFP-10/ESAT-6-specific CD4 T cells in TB disease is characterized by increased expression of Ki67 and CD95 and downregulation of Bcl-2. Expression of Ki67, Bcl-2, CD127, CD57, and CD95 by specific CD3^+^CD8^−^IFN-γ^+^ T cells was measured by flow cytometry, as described in Figure 1. Greater than 90% of CD3^+^CD8^−^IFN-γ^+^ T cells following stimulation with CFP-10 and ESAT-6 peptide pools were found to be CD4^+^ (see Fig. S3), and are referred to here as CD4 T cells. Phenotypic analysis was performed on individuals with positive CD4 T cell responses (defined as CD3^+^CD8^−^IFN-γ^+^) to either CFP-10 or ESAT-6 peptide pools (n = 16/18 with LTBI; n = 10/20 with TB disease). (A) Summary data of the percentage of specific IFN-γ^+^ CD4 T cells expressing Ki67, Bcl-2, CD127, CD57 and CD95 in individuals with LTBI and patients with TB disease. Differences were assessed using the Mann-Whitney test, followed by the Bonferroni correction for multiple comparisons. (B) Flow cytometry data indicating expression of Bcl-2 and Ki67 by ESAT-6-specific CD4 T cells in an individual with LTBI (top plot) and an individual with TB disease (bottom plot). Plots are shown gated on CD3^+^CD8^−^IFN-γ^+^ T cells. (C) Co-expression patterns of Bcl-2, CD95, and Ki67 by IFN-γ^+^ CD4 T cells in individuals with LTBI (open bars; n = 16) and TB disease (grey bars; n = 10). Differences were assessed using the Mann-Whitney test, followed by the Bonferroni correction for multiple comparisons. (D) Comparison of the proportion of Bcl-2^+^CD95^−^Ki67^−^ cells contributing to the total CFP-10/ESAT-6-specific CD4 T cell responses in individuals with LTBI and patients with TB disease. Horizontal lines represent the median. Differences were assessed using the Mann-Whitney test. The dotted line indicates the cut-off (7%) that distinguishes individuals with LTBI and TB disease, with 90% sensitivity and 75% specificity. (E) Receiver operator characteristic (ROC) curve indicating the sensitivity and specificity of the proportion of CFP-10/ESAT-6-specific CD4 T cells that are Bcl-2^+^CD95^−^Ki67^−^ to distinguish individuals with LTBI and TB disease. An area under the ROC curve (AUC) analysis was performed to further evaluate the performance of this phenotypic expression profile in distinguishing individuals with LTBI and TB disease. (F) Co-expression patterns of Bcl-2, CD95, and Ki67 by CMV pp65-specific CD4 T cells in individuals with LTBI (open bars) and TB disease (grey bars). Data are shown from 16 individuals with LTBI and 13 patients with TB disease who had positive CD4 T cell responses to a CMV pp65 peptide pool. No differences were found between individuals with LTBI and TB for any of the CMV-specific CD4 T cell subsets (Mann-Whitney test).

Although distinct patterns of Bcl-2, CD57, and CD95 co-expression by CFP-10/ESAT-6-specific CD8 T cells were highly sensitive and specific in distinguishing latent infection from active disease, these phenotypic measurements can only be analyzed in individuals who have sufficient numbers of specific CD8 T cells to be detectable in blood ex vivo. CFP-10/ESAT-6-specific CD8 T cells are generally less readily detectable in peripheral blood than CFP-10/ESAT-6-specific CD4 T cells, particularly in the setting of LTBI [30]. Therefore, we analyzed expression of each phenotypic marker on CFP-10/ESAT-6-specific CD3^+^CD8^−^IFN-γ^+^ cells, >90% of which were confirmed to be CD4^+^ in a separate ICS assay (Figure S3), and thus referred to here subsequently as CD4 T cells. Similar to CD8 T cells, CFP-10/ESAT-6-specific CD4 T cells expressed significantly higher levels of Ki67 and CD95, and lower levels of Bcl-2, in patients with TB disease, compared with individuals with LTBI (Figure 3A). However, unlike CFP-10/ESAT-6-specific CD8 T cells, expression of CD127 and CD57 by CFP-10/ESAT-6-specific CD4 T cells was not different between individuals with LTBI and patients with active TB disease (Figure 3A). Boolean analysis of Bcl-2, Ki67 and CD95 co-expression by CFP-10/ESAT-6-specific CD4 T cells indicated that Bcl-2^−^CD95^+^Ki67^+^ CD4 T cells were found almost exclusively in patients TB patients, and rarely in individuals with LTBI (Figure 3B-C; Figure S4). TB patients also expressed higher proportions of CFP-10/ESAT-6-specific CD4 T cells with a Bcl-2^−^CD95^+^Ki67^−^ phenotype, whereas individuals with LTBI had greater proportions of Bcl-2^+^ cells, with or without CD95 co-expression, in the absence of Ki67 (Figure 3C-E; Figure S4). No differences in expression of these molecules were found on cytomegalovirus (CMV) pp65-specific CD4 T cells within the same individuals (Figure 3F), thus suggesting the phenotypically distinct subsets of antigen-specific CD4 T cells expressing these combinations of Bcl-2, Ki67, and CD95 are unique to CFP-10/ESAT-6-specific CD4 T cells in TB patients, and not indicative of generalized immune activation and inflammation in the setting of active disease.

### CFP-10/ESAT-6-specific CD8 T cell phenotype is associated with mycobacterial antigen load

**Figure 4:**
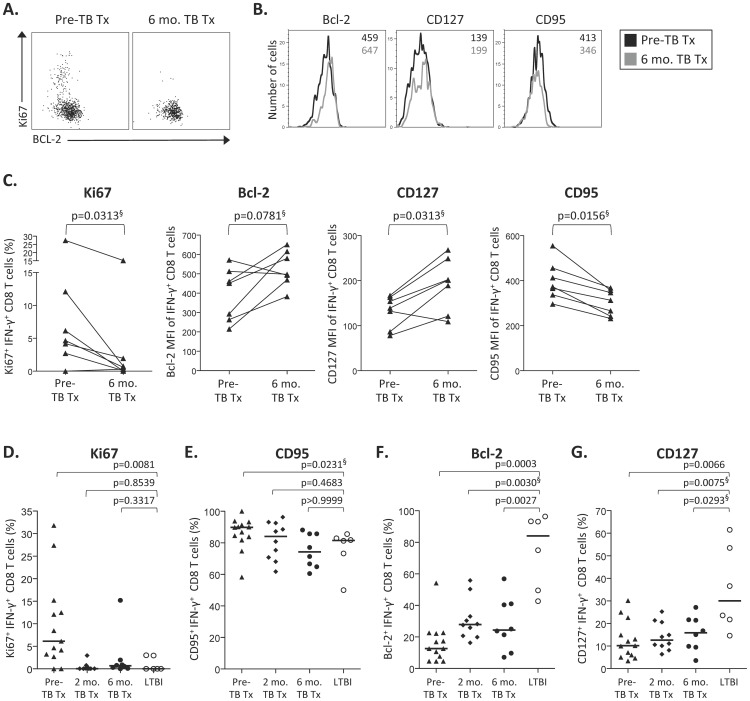
CFP-10/ESAT-6-specific CD8 T cell phenotype is associated with mycobacterial antigen load. Phenotypic analysis of CFP-10 and ESAT-6-specific IFN-γ^+^ CD8 T cells was performed as described in Figure 1. Cells were analyzed prior to initiating anti-TB treatment (Pre-TB Tx), and 2 months and 6 months after initiation of treatment. (**A**) Flow cytometry data indicating Ki67 and Bcl-2 expression by IFN-γ^+^ ESAT-6-specific CD8 T cells from a TB diseased patient at two time points: prior to treatment and 6 months after initiation of treatment, corresponding to the end of the treatment period. (**B**) Representative histogram overlays indicating expression of Bcl-2, CD127 and CD95 within CFP-10/ESAT-6-specific IFN-γ^+^ CD8 T cells. Black lines indicate expression prior to treatment; grey lines indicate expression 6 months after initiation of treatment. The numbers in the upper right corner of the histograms indicate the median fluorescence intensity (MFI) at each time point (black: pre-treatment MFI; grey: 6-month TB treatment MFI). Data shown in panels A and B are gated on VIVID^l^°CD3^+^CD8^+^IFN-γ^+^ cells. (**C**) Summary data comparing the expression of Ki67, Bcl-2, CD127 and CD95 on CFP-10/ESAT-6-specific IFN-γ^+^ CD8 T cells from the same individual at two time points: prior to treatment (Pre-TB Tx) and at the end of the 6-month treatment period (6 mo. TB Tx). Data are shown from 7 individuals who maintained positive CFP-10/ESAT-6-specific CD8 T cell responses throughout the 6-month duration of treatment. Differences between time points were assessed using the Wilcoxon matched pairs test. § indicates p values that did not retain statistical significance after applying the Bonferroni correction for multiple comparisons. The proportion of CFP-10/ESAT-6-specific IFN-γ^+^ CD8 T cells that express Ki67 (**D**), CD95 (**E**), Bcl-2 (**F**), and CD127 (**G**) are shown for individuals with LTBI (n = 6), and TB diseased patients prior to treatment (n = 13; Pre-TB Tx), and 2 months (n = 10; 2 mo. TB Tx) and 6 months (n = 8; 6 mo. TB Tx) following initiation of treatment. Differences in panels D-G were first assessed using a Kruskal-Wallis test, followed by a Dunn's post-test to correct for multiple comparisons; § indicates p values that did not remain significant following correction with the Dunn's post-test.

Co-expression patterns of Bcl-2, CD57 and CD95 by CFP-10/ESAT-6-specific CD8 T cells were significantly different between individuals with LTBI and patients with active TB at the time of diagnosis. We next performed a longitudinal analysis of CFP-10/ESAT-6-specific CD8 T cell phenotype in patients with active TB during the treatment period to further define the relationship between CFP-10/ESAT-6-specific CD8 T cell phenotype and mycobacterial antigen load (Figure 4A-B). By the end of the 6-month TB treatment period, Ki67 and CD95 expression had decreased, whereas Bcl-2 and CD127 expression increased in CFP-10/ESAT-6-specific CD8 T cells, compared with pre-treatment levels (Figure 4C). As all patients had an excellent clinical response to therapy, these data therefore suggest that mycobacterial load drives distinct changes in CFP-10/ESAT-6-specific CD8 T cell phenotype.

To address the kinetics of mycobacterial antigen load-associated changes in CFP-10 and ESAT-6-specific CD8 T cell phenotype, we compared expression of each molecule on CFP-10/ESAT-6-specific CD8 T cells between persons with LTBI, who had not received any treatment, and TB diseased patients at three time points: pre-treatment, and 2 and 6 months following initiation of treatment. After 2 months of treatment, expression of Ki67 and CD95 by CFP-10/ESAT-6-specific CD8 T cells had decreased to similar levels observed in persons with LTBI (Figure 4D–E). By contrast, even after successful completion of the standard course of 6 months of anti-TB treatment, expression of Bcl-2 and CD127 by CFP-10/ESAT-6-specific CD8 T cells of TB disease patients remained significantly lower than in persons with LTBI (Figure 4F–G).

**Figure 5:**
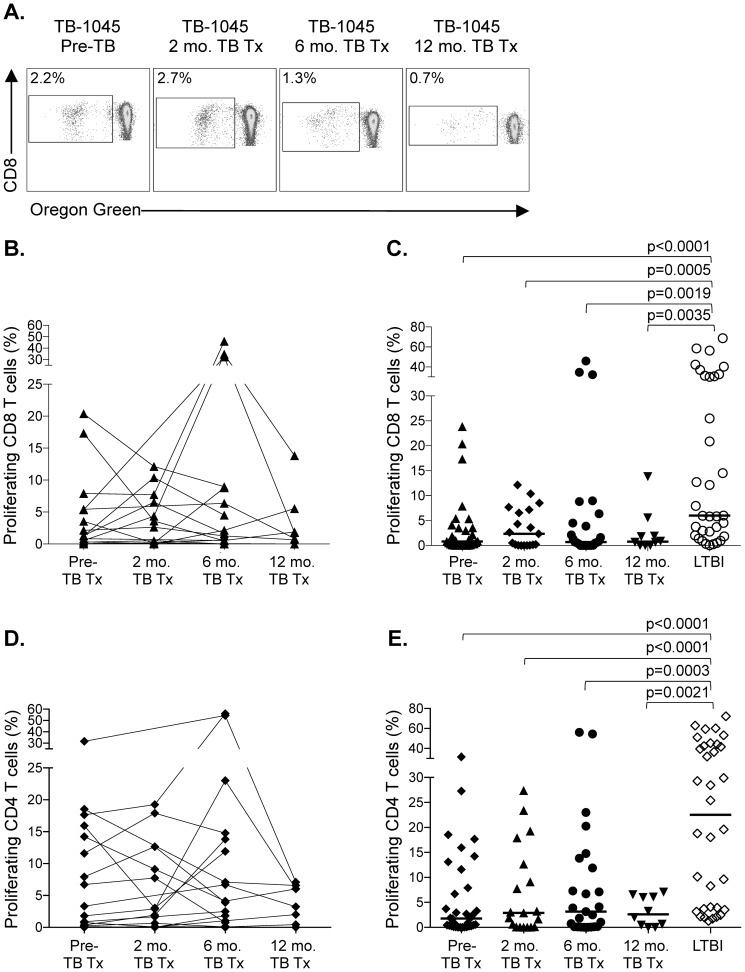
Lack of restoration of CFP-10/ESAT-6-specific CD4 and CD8 T cell proliferative capacity following completion of treatment. The proliferative capacity of CFP-10/ESAT-6-specific CD4 and CD8 T cells was measured following a 6-day stimulation of freshly isolated PBMCs with CFP-10 and ESAT-6 peptide pools. (A) Flow cytometry data of the proliferative capacity of antigen-specific CD8 T cells from a TB diseased patient analyzed at 4 time points: prior to treatment (Pre-TB Tx), 2 months of treatment (2 mo. TB Tx), 6 months of treatment (6 mo. TB Tx, corresponding to the end of the treatment period), and 6 months after completion of treatment (12 mo. TB Tx). The cells shown are gated on VIVID^l^°CD3^+^CD8^+^ lymphocytes. The percentage on each plot indicates the percentage of proliferating CD8 T cells after subtraction of background proliferation in the negative control condition. (B, D) Longitudinal analysis of the proliferative capacity of CFP-10 and ESAT-6-specific CD8 (panel B) and CD4 (panel D) T cells in a subset of TB diseased patients who were followed for up to one year after TB diagnosis (n = 19 with at least 3 time points available for analysis). There were no significant differences in the frequency of proliferating antigen-specific CD8 or CD4 T cells over time (Kruskal-Wallis test). (C, E) Cross-sectional comparison of the CFP-10 and ESAT-6-specific CD8 (panel C) and CD4 (panel E) T cell proliferative capacity between individuals with LTBI (n = 34) and active TB disease at 4 time points (pre-TB tx: n = 34; 2 months of TB tx: n = 19; 6 months of TB Tx [corresponding to the end of the treatment period]: n = 25; 12 months post initiation of TB tx [corresponding to 6 months after the end of the treatment period]: n = 10). Data are shown after subtraction of background proliferation in the negative control condition. Differences in panels C and E were first assessed using a Kruskal-Wallis test, followed by a Dunn's post-test to correct for multiple comparisons. All comparisons remained significant following correction with the Dunn's post-test.

Finally, we investigated whether the longitudinal phenotypic changes in CFP-10 and ESAT-6-specific CD8 T cells were associated with restoration of functional capacity. We therefore measured CFP-10/ESAT-6-specific T cell proliferative capacity (Figure 5A), a functional capacity that is significantly impaired in patients with TB disease [30], in patients before, during, and after treatment for TB disease. There was no evidence of sustained augmentation of CFP-10/ESAT-6-specific CD8 T cell proliferative capacity after completion of treatment (Figure 5B). Moreover, the proliferative capacity of patients with TB disease at all time points analyzed remained significantly lower than in persons with LTBI (Figure 5C). Similar results were found for CFP-10/ESAT-6-specific CD4 T cells (Figure 5D–E), thus suggesting lack of restoration of CD8 T cell proliferative capacity after TB treatment may be associated with impaired CD4 T cell help in patients who develop TB disease. Taken together, these data indicate the phenotypic profile of CFP-10 and ESAT-6-specific CD8 T cells is associated with mycobacterial antigen load; however, despite successful treatment for active TB disease, CFP-10/ESAT-6-specific CD8 T cell function, as measured by proliferative capacity, is not restored.

## Discussion

We characterized phenotypic profiles of human CFP-10 and ESAT-6-specific CD8 T cells that are associated with immune containment of the pathogen – LTBI – and with active pulmonary disease. We made the following observations: 1) In TB diseased persons, CFP-10/ESAT-6-specific CD8 T cells are characterized by a pro-apoptotic phenotype and by increased expression of molecules involved in activation, apoptosis, and senescence, when compared with persons with LTBI; 2) When CFP-10/ESAT-6-specific CD8 T cells are detectable, distinct phenotypic profiles of Bcl-2, CD57, and/or CD95 co-expression are highly specific and sensitive in differentiating TB disease and LTBI; 3) Similar to CD8 T cells, CFP-10/ESAT-6-specific CD4 T cells in patients with TB disease also display a pro-apoptotic phenotype, with increased Ki67 and CD95 expression and decreased Bcl-2 expression; 4) CFP-10/ESAT-6-specific CD8 T cell phenotype is likely to be associated with mycobacterial antigen load, as suggested by rapid changes in expression of specific molecules following initiation of treatment; 5) Despite distinct changes in CFP-10/ESAT-6-specific CD8 T cell phenotype following successful treatment for TB disease, expression of Bcl-2 and CD127 remains lower than levels observed by CFP-10/ESAT-6-specific CD8 T cells in persons with LTBI, thus suggesting greater susceptibility to apoptosis in treated TB patients; neither does CFP-10/ESAT-6-specific CD8 nor CD4 T cell proliferative capacity recover following completion of TB treatment.

Progressive dysfunction of antigen-specific CD8 T cells in chronic viral infections has been well described [58]; however, much less is known about potential CD8 T cell dysfunction in chronic bacterial infections of humans. Inefficient control of Mtb infection in both humans and animal models of infection may be due to marked susceptibility of T cells to apoptosis [59]–[61]. This was supported by our findings that CFP-10/ESAT-6-specific CD8 T cells from TB patients have reduced expression of Bcl-2, a molecule which inhibits programmed cell death by binding to the pro-apoptotic proteins Bax and Bak [62]. We also showed upregulation of CD95 by CFP-10/ESAT-6-specific CD8 T cells of patients with TB; susceptibility to apoptosis has previously been linked to downregulation of Bcl-2 in conjunction with upregulation of CD95 (Fas) [51]–[53], [63]. Moreover, suboptimal immunity to another intracellular human pathogen, *Trypanosoma cruzi*, has been associated with generation of specific CD8 T cells that lack proliferative capacity and simultaneously express high levels of CD95 [64]. Thus, our data from TB diseased patients are consistent with generation of suboptimal CD8 T cell immunity and a pro-apoptotic T cell phenotype, similar to that previously described in other chronic infections. Future studies utilizing MHC class I tetramers will be necessary to determine whether apoptosis is directly induced in CFP-10/ESAT-6-specific CD8 T cells following stimulation with cognate antigen, or whether these cells persist in a functionally inactive state.

Our phenotypic analysis of CD8 T cells also revealed distinct patterns of expression of phenotypic molecules in individuals with LTBI and patients with TB disease: CFP-10/ESAT-6-specific CD8 T cells in persons with LTBI co-expressed Bcl-2 and CD95 and did not express CD57, whereas patients with TB disease expressed CD95, with or without CD57 co-expression, but expressed very low levels of Bcl-2. It is important to note that this phenotypic signature is particular to CFP-10 and ESAT-6-specific CD8 T cells, which were detectable ex vivo by our ICS assay in 33% of persons with LTBI and 65% of patients with TB disease. However, we also observed significant differences in expression patterns of Bcl-2, CD95, and Ki67 by CFP-10 and ESAT-6-specific CD3^+^CD8^−^IFN-γ^+^ cells (confirmed in a separate ICS assay to be CD4 T cells) in individuals with LTBI and patients with TB disease, thus suggesting these phenotypic markers are also differentially expressed by antigen-specific CD4 T cells in the context of latent infection and active disease in humans. These findings provide proof of concept that phenotypic signatures of antigen-specific T cells may be further developed to correctly classify individuals with different stages of Mtb infection and disease with high degrees of sensitivity and specificity. It is important to note in this study that we used IFN-γ as a read-out of antigen specificity, and thus the phenotype of antigen-specific T cells that produce other effector molecules, in the absence of IFN-γ, remains unknown. Moreover, we focused on T cell responses specific for epitopes in the immunodominant antigens CFP-10 and ESAT-6, and it is possible that T cells specific for other antigens in Mtb may display different phenotypic signatures than observed in this study. Additional studies of larger cohorts of individuals are required to further define antigen-specific T cell phenotypic signatures that are associated with different Mtb infection and disease states, and to assess the utility of Mtb-specific T cell phenotypic signatures in predicting risk of TB disease prospectively.

Within two months of treatment, CFP-10/ESAT-6-specific CD8 T cell expression of Ki67 and CD95 in TB patients decreased to similar levels observed in persons with LTBI, suggesting that expression of particular markers by CFP-10/ESAT-6-specific T cells may be an indication of bacterial load levels. CFP-10/ESAT-6-specific CD8 T cell expression of Bcl-2 and CD127 also increased during treatment, but expression never decreased to levels observed in persons with LTBI. It is important to note here that individuals with positive tuberculin skin tests (TST) and/or IFN-γ release assays (IGRA) likely represent a heterogeneous population encompassing a spectrum of bacterial loads, ranging from eradication of bacteria to persistence of bacteria replicating at low levels in the absence of clinical symptoms [65], [66]. The dynamic nature of LTBI is further evidenced by imaging studies in Mtb-infected cynomolgous macaques, which indicate high levels of variability of bacterial replication and bacterial killing in individual granulomas within a given host [67]. Thus the phenotype of CFP-10/ESAT-6-specific CD8 T cells we observed in individuals with LTBI likely represents a snapshot of antigen-specific CD8 T cell phenotypes, which may change over time given the potential for fluctuations in the levels of bacterial replication in individuals with persistent Mtb infection.

Given that expression of CD127, the IL-7 receptor α chain, is important for T cell survival and homeostasis [68], [69], and that expression of Bcl-2 is important for preventing apoptosis [62], the relatively lower levels of CD127 and Bcl-2 expression on CFP-10/ESAT-6-specific CD8 T cells in patients treated for TB, compared with individuals with LTBI, suggests antigen-specific memory CD8 T cells may remain more prone to cell death in patients following successful treatment of TB disease. Importantly, we also found no evidence of restoration of CFP-10/ESAT-6-specific CD4 or CD8 T cell proliferative capacity, even in persons evaluated up to 12 months after diagnosis and successful completion of treatment. These data suggest memory CD4 and CD8 T cells with impaired functional capacity persist in individuals successfully treated for TB disease. It is tempting to speculate that compromised memory T cell function is one mechanism underlying the risk of recurrent TB disease, which can occur in up to 20% of HIV-uninfected and one-third of HIV-infected persons in South Africa [70].

In summary, our findings further define parameters of antigen-specific CD8 T cells that are associated with successful immune control of Mtb infection (LTBI) and development of active TB disease. Moreover, the data presented here provide a platform for future prospective, longitudinal studies to evaluate phenotypic and functional signatures of CFP-10/ESAT-6-specific T cells that may differentiate individuals at different stages of infection. Finally, these data provide novel insights regarding immune parameters, such as inadequate generation of long-term memory T cells and lack of proliferative capacity, which may be associated with susceptibility to recurrent episodes of active TB disease.

## Supporting Information

Figure S1 **Flow cytometry gating strategies used in phenotypic analysis of CFP-10/ESAT-6-specific CD8 T cells.** PBMCs were thawed and stimulated with antigen as described in Figure 1. (**A**) Doublet cell populations were excluded using a forward scatter area (FSC-A) versus forward scatter height (FSC-H) plot. Lymphocytes were selected based on FSC-A and side scatter area (SSC-A) characteristics. Viable T cells were defined as VIVID^l^°CD3^+^ cells. (**B**) CD8^+^ cells were further selected within the viable T cell gate, and analyzed for expression of IFN-γ, CD57, Bcl-2, Ki67, CD95, and CD127. Representative data from SEB-stimulated PBMCs from a patient with TB disease are shown.
(PDF)

Figure S2 **The proportion of CFP-10/ESAT-6-specific CD8 T cells that are Bcl-2^−^CD57^+^CD95^+^ and Bcl-2^+^CD57^−^CD95^−^ differentiates individuals with LTBI and TB disease.** Co-expression patterns of Bcl-2, CD57, and CD95 on CFP-10/ESAT-6-specific CD8 T cells were determined as described in Figure 2. (**A**) Comparison of the proportion of Bcl-2^−^CD57^+^CD95^+^ cells contributing to the total CFP-10/ESAT-6-specific CD8 T cell response in individuals with LTBI and patients with TB disease. The dotted line indicates the cut-off (7%) that distinguishes individuals with LTBI and patients with TB disease, with 92% sensitivity and 100% specificity. An ROC curve is shown indicating the sensitivity and specificity of the proportion of CFP-10/ESAT-6-specific CD8 T cells that are Bcl-2^−^CD57^+^CD95^+^ in distinguishing individuals with LTBI and patients with TB disease. (**B**) Comparison of the proportion of Bcl-2^+^CD57^−^CD95^−^ cells contributing to the total CFP-10/ESAT-6-specific CD8 T cell response in individuals with LTBI and patients with TB disease. The dotted line indicates the cut-off (3.3%) that distinguishes individuals with LTBI and patients with TB disease, with 92% sensitivity and 100% specificity. An ROC curve is shown indicating the sensitivity and specificity of the proportion of CFP-10/ESAT-6-specific CD8 T cells that are Bcl-2^+^CD57^−^CD95^−^ in distinguishing individuals with LTBI and patients with TB disease. An area under the ROC curve (AUC) analysis was performed to further evaluate the performance of these particular phenotypic expression profiles in distinguishing individuals with LTBI and patients with TB disease.
(PDF)

Figure S3 **The majority of CFP-10 and ESAT-6-specific CD3^+^CD8^−^IFN-γ^+^ T cells are CD4^+^.** PBMCs from individuals with LTBI and patients with TB disease were stimulated with CFP-10 and ESAT-6 peptide pools for 6 hours as described in the Materials and Methods section. Cells were stained with LIVE/DEAD Fixable Violet Dead Cell Stain (ViVid), anti-CD3 allophycocyanin-H7 (SK7), anti-IFN-γ Alexa Fluor 700 (B27), anti-CD8 PerCP-Cy5.5 (SK-1), all from BD Biosciences, and anti-CD4 QDot605 (S3.5) from Life Technologies. (**A**) Flow cytometry data representing the gating strategy for the analysis of CD4 expression on live CD3^+^CD8^−^IFN-γ^+^ T cells. Data are shown for PBMCs stimulated with CFP-10 peptide pool from a patient with TB disease (top row) and an individual with LTBI (bottom row). (**B**) Composite data indicating the percentage of CD3^+^CD8^−^IFN-γ^+^ T cells that are CD4^+^ in individuals with LTBI (n = 9) and patients with TB disease (n = 5). Each data point represents a single individual; colors indicate the antigen specificity of the response measured. (**C**) Flow cytometry data indicating the gating strategy used for phenotypic analysis of VIVID^l^°CD3^+^CD8^−^IFN-γ^+^ cells. ESAT-6-specific IFN-γ^+^ cells from an individual with LTBI are shown as black dots overlayed on the total VIVID^l^°CD3^+^CD8^−^ population.
(PDF)

Figure S4 **Predictive values of Bcl-2, CD95, and Ki67 expression by CFP-10/ESAT-6-specific CD4 T cells in distinguishing individuals with LTBI from TB disease patients.** Co-expression patterns of Bcl-2, CD95, and Ki67 on CFP-10/ESAT-6-specific CD4 T cells were determined as described in Figure 3. (**A**) Comparison of the proportion of Bcl-2^−^CD95^+^Ki67^+^ cells contributing to the total CFP-10/ESAT-6-specific CD4 T cell response in individuals with LTBI and TB disease patients. The dotted line indicates the cut-off (7%) that distinguishes individuals with LTBI and patients with TB disease, with 80% sensitivity and 100% specificity. An ROC curve is shown indicating the sensitivity and specificity of the proportion of CFP-10/ESAT-6-specific CD4 T cells that are Bcl-2^−^CD95^+^Ki67^+^ in distinguishing individuals with LTBI and TB disease patients. (**B**) Comparison of the proportion of Bcl-2^+^CD95^+^Ki67^−^ cells contributing to the total CFP-10/ESAT-6-specific CD4 T cell response in individuals with LTBI and TB disease patients. The dotted line indicates the cut-off (27%) that distinguishes individuals with LTBI from TB disease patients, with 80% sensitivity and 81% specificity. An ROC curve is shown indicating the sensitivity and specificity of the proportion of CFP-10/ESAT-6-specific CD4 T cells that are Bcl-2^+^CD95^+^Ki67^−^ in distinguishing individuals with LTBI and TB disease patients. (**C**) Comparison of the proportion of Bcl-2^−^CD95^+^Ki67^−^ cells contributing to the total CFP-10/ESAT-6-specific CD4 T cell response in individuals with LTBI and TB disease patients. The dotted line indicates the cut-off (44%) that distinguishes individuals with LTBI and patients with TB disease, with 80% sensitivity and 81% specificity. An ROC curve is shown indicating the sensitivity and specificity of the proportion of CFP-10/ESAT-6-specific CD4 T cells that are Bcl-2^−^CD95^+^Ki67^−^ in distinguishing individuals with LTBI and TB disease patients. For panels A, B, and C, an area under the ROC curve (AUC) analysis was performed to further evaluate the performance of these particular phenotypic expression profiles in distinguishing individuals with LTBI from TB disease patients.
(PDF)

## References

[1] Global Tuberculosis Report 2012. World Health Organization. Geneva, Switzerland. Available: http://www.who.int/tb/publications/global_report/en/. Accessed 2013 May 1.

[2] Caruso AM, Serbina N, Klein E, Triebold K, Bloom BR, et al (1999) Mice deficient in CD4 T cells have only transiently diminished levels of IFN-gamma, yet succumb to tuberculosis. J Immunol 162: 5407–5416. 10228018

[3] Scanga CA, Mohan VP, Yu K, Joseph H, Tanaka K, et al (2000) Depletion of CD4(+) T cells causes reactivation of murine persistent tuberculosis despite continued expression of interferon gamma and nitric oxide synthase 2. J Exp Med 192: 347–358. 10934223 10.1084/jem.192.3.347 PMC2193220

[4] Flory CM, Hubbard RD, Collins FM (1992) Effects of in vivo T lymphocyte subset depletion on mycobacterial infections in mice. J Leukoc Biol 51: 225–229. 1347311 10.1002/jlb.51.3.225

[5] Leveton C, Barnass S, Champion B, Lucas S, De Souza B, et al (1989) T-cell-mediated protection of mice against virulent *Mycobacterium tuberculosis* . Infect Immun 57: 390–395. 2492259 10.1128/iai.57.2.390-395.1989 PMC313109

[6] Chan J, Xing Y, Magliozzo RS, Bloom BR (1992) Killing of virulent *Mycobacterium tuberculosis* by reactive nitrogen intermediates produced by activated murine macrophages. J Exp Med 175: 1111–1122. 1552282 10.1084/jem.175.4.1111 PMC2119182

[7] MacMicking JD, North RJ, LaCourse R, Mudgett JS, Shah SK, et al (1997) Identification of nitric oxide synthase as a protective locus against tuberculosis. Proc Natl Acad Sci U S A 94: 5243–5248. 9144222 10.1073/pnas.94.10.5243 PMC24663

[8] MacMicking JD, Taylor GA, McKinney JD (2003) Immune control of tuberculosis by IFN-gamma-inducible LRG-47. Science 302: 654–659. 14576437 10.1126/science.1088063

[9] Bruns H, Meinken C, Schauenberg P, Harter G, Kern P, et al (2009) Anti-TNF immunotherapy reduces CD8+ T cell-mediated antimicrobial activity against *Mycobacterium tuberculosis* in humans. J Clin Invest 119: 1167–1177. 19381021 10.1172/JCI38482 PMC2673881

[10] Stegelmann F, Bastian M, Swoboda K, Bhat R, Kiessler V, et al (2005) Coordinate expression of CC chemokine ligand 5, granulysin, and perforin in CD8+ T cells provides a host defense mechanism against *Mycobacterium tuberculosis* . J Immunol 175: 7474–7483. 16301655 10.4049/jimmunol.175.11.7474

[11] Stenger S, Hanson DA, Teitelbaum R, Dewan P, Niazi KR, et al (1998) An antimicrobial activity of cytolytic T cells mediated by granulysin. Science 282: 121–125. 9756476 10.1126/science.282.5386.121

[12] Brookes RH, Pathan AA, McShane H, Hensmann M, Price DA, et al (2003) CD8+ T cell-mediated suppression of intracellular *Mycobacterium tuberculosis* growth in activated human macrophages. Eur J Immunol 33: 3293–3302. 14635037 10.1002/eji.200324109

[13] Carranza C, Juarez E, Torres M, Ellner JJ, Sada E, et al (2006) *Mycobacterium tuberculosis* growth control by lung macrophages and CD8 cells from patient contacts. Am J Respir Crit Care Med 173: 238–245. 16210664 10.1164/rccm.200503-411OC PMC2662991

[14] Flynn JL, Goldstein MM, Triebold KJ, Koller B, Bloom BR (1992) Major histocompatibility complex class I-restricted T cells are required for resistance to *Mycobacterium tuberculosis* infection. Proc Natl Acad Sci U S A 89: 12013–12017. 1465432 10.1073/pnas.89.24.12013 PMC50688

[15] van Pinxteren LA, Cassidy JP, Smedegaard BH, Agger EM, Andersen P (2000) Control of latent *Mycobacterium tuberculosis* infection is dependent on CD8 T cells. Eur J Immunol 30: 3689–3698. 11169412 10.1002/1521-4141(200012)30:12<3689::AID-IMMU3689>3.0.CO;2-4

[16] Sousa AO, Mazzaccaro RJ, Russell RG, Lee FK, Turner OC, et al (2000) Relative contributions of distinct MHC class I-dependent cell populations in protection to tuberculosis infection in mice. Proc Natl Acad Sci U S A 97: 4204–4208. 10760288 10.1073/pnas.97.8.4204 PMC18197

[17] Mogues T, Goodrich ME, Ryan L, LaCourse R, North RJ (2001) The relative importance of T cell subsets in immunity and immunopathology of airborne *Mycobacterium tuberculosis* infection in mice. J Exp Med 193: 271–280. 11157048 10.1084/jem.193.3.271 PMC2195922

[18] Chen CY, Huang D, Wang RC, Shen L, Zeng G, et al (2009) A critical role for CD8 T cells in a nonhuman primate model of tuberculosis. PLoS Pathog 5: e1000392. 19381260 10.1371/journal.ppat.1000392 PMC2663842

[19] Caccamo N, Meraviglia S, La Mendola C, Guggino G, Dieli F, et al (2006) Phenotypical and functional analysis of memory and effector human CD8 T cells specific for mycobacterial antigens. J Immunol 177: 1780–1785. 16849488 10.4049/jimmunol.177.3.1780

[20] Cho S, Mehra V, Thoma-Uszynski S, Stenger S, Serbina N, et al (2000) Antimicrobial activity of MHC class I-restricted CD8+ T cells in human tuberculosis. Proc Natl Acad Sci U S A 97: 12210–12215. 11035787 10.1073/pnas.210391497 PMC17320

[21] Klein MR, Smith SM, Hammond AS, Ogg GS, King AS, et al (2001) HLA-B*35-restricted CD8 T cell epitopes in the antigen 85 complex of *Mycobacterium tuberculosis* . J Infect Dis 183: 928–934. 11237810 10.1086/319267

[22] Lalvani A, Brookes R, Wilkinson RJ, Malin AS, Pathan AA, et al (1998) Human cytolytic and interferon gamma-secreting CD8+ T lymphocytes specific for *Mycobacterium tuberculosis* . Proc Natl Acad Sci U S A 95: 270–275. 9419365 10.1073/pnas.95.1.270 PMC18198

[23] Lewinsohn DA, Winata E, Swarbrick GM, Tanner KE, Cook MS, et al (2007) Immunodominant tuberculosis CD8 antigens preferentially restricted by HLA-B. PLoS Pathog 3: 1240–1249. 17892322 10.1371/journal.ppat.0030127 PMC2323292

[24] Lewinsohn DM, Swarbrick GM, Cansler ME, Null MD, Rajaraman V, et al (2013) Human CD8 T Cell Antigens/Epitopes Identified by a Proteomic Peptide Library. PLoS One 8: e67016. 23805289 10.1371/journal.pone.0067016 PMC3689843

[25] Lewinsohn DM, Zhu L, Madison VJ, Dillon DC, Fling SP, et al (2001) Classically restricted human CD8+ T lymphocytes derived from *Mycobacterium tuberculosis*-infected cells: definition of antigenic specificity. J Immunol 166: 439–446. 11123322 10.4049/jimmunol.166.1.439

[26] Pathan AA, Wilkinson KA, Wilkinson RJ, Latif M, McShane H, et al (2000) High frequencies of circulating IFN-gamma-secreting CD8 cytotoxic T cells specific for a novel MHC class I-restricted Mycobacterium tuberculosis epitope in *M. tuberculosis*-infected subjects without disease. Eur J Immunol 30: 2713–2721. 11009107 10.1002/1521-4141(200009)30:9<2713::AID-IMMU2713>3.0.CO;2-4

[27] Shams H, Klucar P, Weis SE, Lalvani A, Moonan PK, et al (2004) Characterization of a *Mycobacterium tuberculosis* peptide that is recognized by human CD4+ and CD8+ T cells in the context of multiple HLA alleles. J Immunol 173: 1966–1977. 15265931 10.4049/jimmunol.173.3.1966

[28] Caccamo N, Guggino G, Meraviglia S, Gelsomino G, Di Carlo P, et al (2009) Analysis of *Mycobacterium tuberculosis*-specific CD8 T-cells in patients with active tuberculosis and in individuals with latent infection. PLoS One 4: e5528. 19436760 10.1371/journal.pone.0005528 PMC2678250

[29] Tang ST, van Meijgaarden KE, Caccamo N, Guggino G, Klein MR, et al (2011) Genome-based in silico identification of new *Mycobacterium tuberculosis* antigens activating polyfunctional CD8+ T cells in human tuberculosis. J Immunol 186: 1068–1080. 21169544 10.4049/jimmunol.1002212

[30] Day CL, Abrahams DA, Lerumo L, Janse van Rensburg E, Stone L, et al (2011) Functional Capacity of *Mycobacterium tuberculosis*-Specific T Cell Responses in Humans Is Associated with Mycobacterial Load. J Immunol 187: 2222–2232. 21775682 10.4049/jimmunol.1101122 PMC3159795

[31] Harari A, Rozot V, Enders FB, Perreau M, Stalder JM, et al (2011) Dominant TNF-alpha(+) *Mycobacterium tuberculosis*-specific CD4(+) T cell responses discriminate between latent infection and active disease. Nat Med 17: 372–376. 21336285 10.1038/nm.2299 PMC6570988

[32] Rozot V, Vigano S, Mazza-Stalder J, Idrizi E, Day CL, et al (2013) *Mycobacterium tuberculosis*-specific CD8 T cells are functionally and phenotypically different between latent infection and active disease. Eur J Immunol 43: 1568–1577. 23456989 10.1002/eji.201243262 PMC6535091

[33] Smith SM, Klein MR, Malin AS, Sillah J, Huygen K, et al (2000) Human CD8(+) T cells specific for *Mycobacterium tuberculosis* secreted antigens in tuberculosis patients and healthy BCG-vaccinated controls in The Gambia. Infect Immun 68: 7144–7148. 11083843 10.1128/iai.68.12.7144-7148.2000 PMC97828

[34] Andersson J, Samarina A, Fink J, Rahman S, Grundstrom S (2007) Impaired expression of perforin and granulysin in CD8+ T cells at the site of infection in human chronic pulmonary tuberculosis. Infect Immun 75: 5210–5222. 17664265 10.1128/IAI.00624-07 PMC2168267

[35] Govender L, Abel B, Hughes EJ, Scriba TJ, Kagina BM, et al (2010) Higher human CD4 T cell response to novel *Mycobacterium tuberculosis* latency associated antigens Rv2660 and Rv2659 in latent infection compared with tuberculosis disease. Vaccine 29: 51–57. 20974305 10.1016/j.vaccine.2010.10.022 PMC3376751

[36] Wherry EJ (2011) T cell exhaustion. Nat Immunol 12: 492–499. 21739672 10.1038/ni.2035

[37] Jones RB, Ndhlovu LC, Barbour JD, Sheth PM, Jha AR, et al (2008) Tim-3 expression defines a novel population of dysfunctional T cells with highly elevated frequencies in progressive HIV-1 infection. J Exp Med 205: 2763–2779. 19001139 10.1084/jem.20081398 PMC2585847

[38] Qiu Y, Chen J, Liao H, Zhang Y, Wang H, et al (2012) Tim-3-expressing CD4+ and CD8+ T cells in human tuberculosis (TB) exhibit polarized effector memory phenotypes and stronger anti-TB effector functions. PLoS Pathog 8: e1002984. 23144609 10.1371/journal.ppat.1002984 PMC3493466

[39] Cui W, Kaech SM (2010) Generation of effector CD8+ T cells and their conversion to memory T cells. Immunol Rev 236: 151–166. 20636815 10.1111/j.1600-065X.2010.00926.x PMC4380273

[40] Harari A, Dutoit V, Cellerai C, Bart PA, Du Pasquier RA, et al (2006) Functional signatures of protective antiviral T-cell immunity in human virus infections. Immunol Rev 211: 236–254. 16824132 10.1111/j.0105-2896.2006.00395.x

[41] Zhang N, Bevan MJ (2011) CD8(+) T cells: foot soldiers of the immune system. Immunity 35: 161–168. 21867926 10.1016/j.immuni.2011.07.010 PMC3303224

[42] Yi JS, Cox MA, Zajac AJ (2010) T-cell exhaustion: characteristics, causes and conversion. Immunology 129: 474–481. 20201977 10.1111/j.1365-2567.2010.03255.x PMC2842494

[43] Crawley AM, Angel JB (2012) The influence of HIV on CD127 expression and its potential implications for IL-7 therapy. Semin Immunol 24: 231–240. 22421574 10.1016/j.smim.2012.02.006

[44] Golden-Mason L, Burton JR Jr, Castelblanco N, Klarquist J, Benlloch S, et al (2006) Loss of IL-7 receptor alpha-chain (CD127) expression in acute HCV infection associated with viral persistence. Hepatology 44: 1098–1109. 17058243 10.1002/hep.21365

[45] Radziewicz H, Ibegbu CC, Fernandez ML, Workowski KA, Obideen K, et al (2007) Liver-infiltrating lymphocytes in chronic human hepatitis C virus infection display an exhausted phenotype with high levels of PD-1 and low levels of CD127 expression. J Virol 81: 2545–2553. 17182670 10.1128/JVI.02021-06 PMC1865979

[46] Barber DL, Wherry EJ, Masopust D, Zhu B, Allison JP, et al (2006) Restoring function in exhausted CD8 T cells during chronic viral infection. Nature 439: 682–687. 16382236 10.1038/nature04444

[47] Day CL, Kaufmann DE, Kiepiela P, Brown JA, Moodley ES, et al (2006) PD-1 expression on HIV-specific T cells is associated with T-cell exhaustion and disease progression. Nature 443: 350–354. 16921384 10.1038/nature05115

[48] Hockenbery D, Nunez G, Milliman C, Schreiber RD, Korsmeyer SJ (1990) Bcl-2 is an inner mitochondrial membrane protein that blocks programmed cell death. Nature 348: 334–336. 2250705 10.1038/348334a0

[49] Appay V, Dunbar PR, Callan M, Klenerman P, Gillespie GM, et al (2002) Memory CD8+ T cells vary in differentiation phenotype in different persistent virus infections. Nat Med 8: 379–385. 11927944 10.1038/nm0402-379

[50] Grayson JM, Harrington LE, Lanier JG, Wherry EJ, Ahmed R (2002) Differential sensitivity of naive and memory CD8+ T cells to apoptosis in vivo. J Immunol 169: 3760–3770. 12244170 10.4049/jimmunol.169.7.3760

[51] Boudet F, Lecoeur H, Gougeon ML (1996) Apoptosis associated with ex vivo down-regulation of Bcl-2 and up-regulation of Fas in potential cytotoxic CD8+ T lymphocytes during HIV infection. J Immunol 156: 2282–2293. 8690919

[52] Slyker JA, Rowland-Jones SL, Dong T, Reilly M, Richardson B, et al (2012) Acute cytomegalovirus infection is associated with increased frequencies of activated and apoptosis-vulnerable T cells in HIV-1-infected infants. J Virol 86: 11373–11379. 22875969 10.1128/JVI.00790-12 PMC3457128

[53] Yoshino T, Kondo E, Cao L, Takahashi K, Hayashi K, et al (1994) Inverse expression of bcl-2 protein and Fas antigen in lymphoblasts in peripheral lymph nodes and activated peripheral blood T and B lymphocytes. Blood 83: 1856–1861. 7511441

[54] Brenchley JM, Karandikar NJ, Betts MR, Ambrozak DR, Hill BJ, et al (2003) Expression of CD57 defines replicative senescence and antigen-induced apoptotic death of CD8+ T cells. Blood 101: 2711–2720. 12433688 10.1182/blood-2002-07-2103

[55] Ibegbu CC, Xu YX, Harris W, Maggio D, Miller JD, et al (2005) Expression of killer cell lectin-like receptor G1 on antigen-specific human CD8+ T lymphocytes during active, latent, and resolved infection and its relation with CD57. J Immunol 174: 6088–6094. 15879103 10.4049/jimmunol.174.10.6088

[56] Papagno L, Spina CA, Marchant A, Salio M, Rufer N, et al (2004) Immune activation and CD8+ T-cell differentiation towards senescence in HIV-1 infection. PLoS Biol 2: E20. 14966528 10.1371/journal.pbio.0020020 PMC340937

[57] Roederer M, Nozzi JL, Nason MX (2011) SPICE: Exploration and analysis of post-cytometric complex multivariate datasets. Cytometry A 79: 167–174. 21265010 10.1002/cyto.a.21015 PMC3072288

[58] Shin H, Wherry EJ (2007) CD8 T cell dysfunction during chronic viral infection. Curr Opin Immunol 19: 408–415. 17656078 10.1016/j.coi.2007.06.004

[59] Hirsch CS, Johnson JL, Okwera A, Kanost RA, Wu M, et al (2005) Mechanisms of apoptosis of T-cells in human tuberculosis. J Clin Immunol 25: 353–364. 16133992 10.1007/s10875-005-4841-4

[60] Hirsch CS, Toossi Z, Vanham G, Johnson JL, Peters P, et al (1999) Apoptosis and T cell hyporesponsiveness in pulmonary tuberculosis. J Infect Dis 179: 945–953. 10068591 10.1086/314667

[61] Kremer L, Estaquier J, Wolowczuk I, Biet F, Ameisen JC, et al (2000) Ineffective cellular immune response associated with T-cell apoptosis in susceptible *Mycobacterium bovis* BCG-infected mice. Infect Immun 68: 4264–4273. 10858244 10.1128/iai.68.7.4264-4273.2000 PMC101741

[62] Kale J, Liu Q, Leber B, Andrews DW (2012) Shedding light on apoptosis at subcellular membranes. Cell 151: 1179–1184. 23217705 10.1016/j.cell.2012.11.013

[63] Wang XZ, Stepp SE, Brehm MA, Chen HD, Selin LK, et al (2003) Virus-specific CD8 T cells in peripheral tissues are more resistant to apoptosis than those in lymphoid organs. Immunity 18: 631–642. 12753740 10.1016/s1074-7613(03)00116-x

[64] Vasconcelos JR, Bruna-Romero O, Araujo AF, Dominguez MR, Ersching J, et al (2012) Pathogen-induced proapoptotic phenotype and high CD95 (Fas) expression accompany a suboptimal CD8+ T-cell response: reversal by adenoviral vaccine. PLoS Pathog 8: e1002699. 22615561 10.1371/journal.ppat.1002699 PMC3355083

[65] Barry CE 3rd, Boshoff HI, Dartois V, Dick T, Ehrt S, et al (2009) The spectrum of latent tuberculosis: rethinking the biology and intervention strategies. Nat Rev Microbiol 7: 845–855. 19855401 10.1038/nrmicro2236 PMC4144869

[66] Lin PL, Flynn JL (2010) Understanding latent tuberculosis: a moving target. J Immunol 185: 15–22. 20562268 10.4049/jimmunol.0903856 PMC3311959

[67] Lin PL, Ford CB, Coleman MT, Myers AJ, Gawande R, et al (2014) Sterilization of granulomas is common in active and latent tuberculosis despite within-host variability in bacterial killing. Nat Med 20: 75–79. 24336248 10.1038/nm.3412 PMC3947310

[68] Mazzucchelli R, Durum SK (2007) Interleukin-7 receptor expression: intelligent design. Nat Rev Immunol 7: 144–154. 17259970 10.1038/nri2023

[69] Schluns KS, Kieper WC, Jameson SC, Lefrancois L (2000) Interleukin-7 mediates the homeostasis of naive and memory CD8 T cells in vivo. Nat Immunol 1: 426–432. 11062503 10.1038/80868

[70] Glynn JR, Murray J, Bester A, Nelson G, Shearer S, et al (2010) High rates of recurrence in HIV-infected and HIV-uninfected patients with tuberculosis. J Infect Dis 201: 704–711. 20121434 10.1086/650529

