# Supplementary figures and images for: Patients with Tuberculosis Disease Have Mycobacterium tuberculosis-Specific CD8 T Cells with a Pro-Apoptotic Phenotype and Impaired Proliferative Capacity, Which Is Not Restored following Treatment
Source: PLoS One. 2014 Apr 16;9(4):e94949. doi: 10.1371/journal.pone.0094949 (PMC3989259; doi:10.1371/journal.pone.0094949)

Figure S1

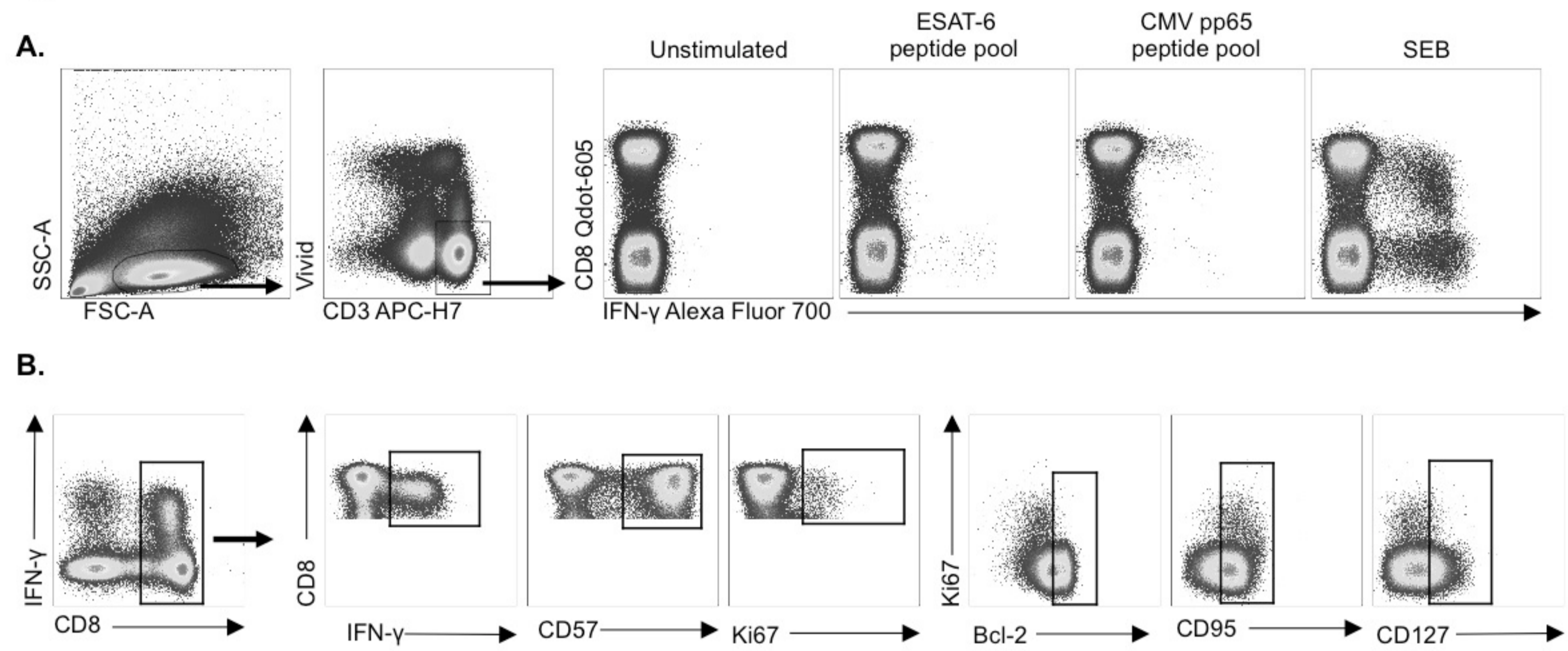

Supplement: Figure S1 — Flow cytometry gating strategies used in phenotypic analysis of CFP-10/ESAT-6-specific CD8 T cells. PBMCs were thawed and stimulated with antigen as described in Figure 1. (A) Doublet cell populations were excluded using a forward scatter area (FSC-A) versus forward scatter height (FSC-H) plot. Lymphocytes were selected based on FSC-A and side scatter area (SSC-A) characteristics. Viable T cells were defined as VIVIDl°CD3+ cells. (B) CD8+ cells were further selected within the viable T cell gate, and analyzed for expression of IFN-γ, CD57, Bcl-2, Ki67, CD95, and CD127. Representative data from SEB-stimulated PBMCs from a patient with TB disease are shown. (PDF) [file pone.0094949.s001.pdf]

Figure S2

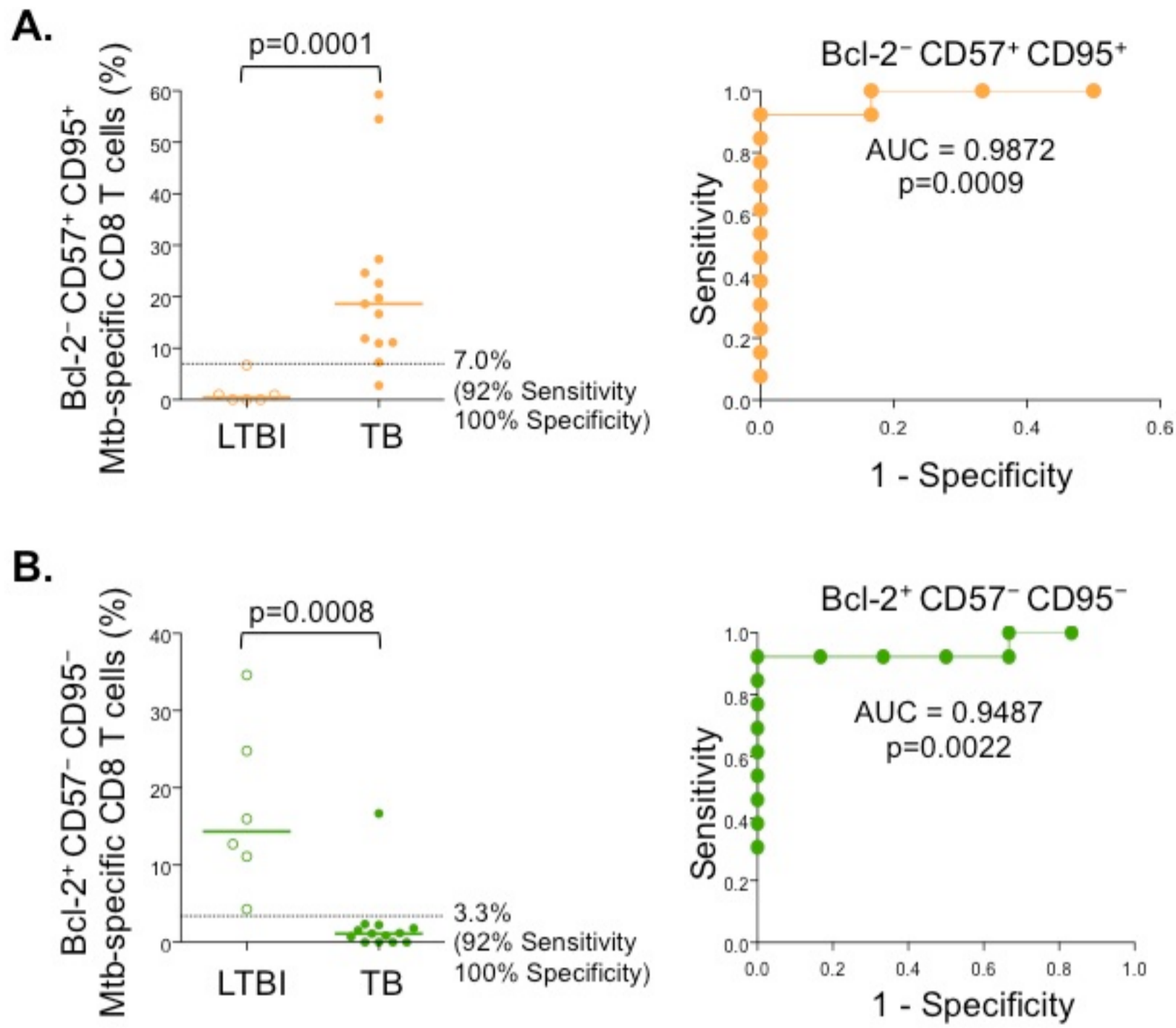

Supplement: Figure S2 — The proportion of CFP-10/ESAT-6-specific CD8 T cells that are Bcl-2−CD57+CD95+ and Bcl-2+CD57−CD95− differentiates individuals with LTBI and TB disease. Co-expression patterns of Bcl-2, CD57, and CD95 on CFP-10/ESAT-6-specific CD8 T cells were determined as described in Figure 2. (A) Comparison of the proportion of Bcl-2−CD57+CD95+ cells contributing to the total CFP-10/ESAT-6-specific CD8 T cell response in individuals with LTBI and patients with TB disease. The dotted line indicates the cut-off (7%) that distinguishes individuals with LTBI and patients with TB disease, with 92% sensitivity and 100% specificity. An ROC curve is shown indicating the sensitivity and specificity of the proportion of CFP-10/ESAT-6-specific CD8 T cells that are Bcl-2−CD57+CD95+ in distinguishing individuals with LTBI and patients with TB disease. (B) Comparison of the proportion of Bcl-2+CD57−CD95− cells contributing to the total CFP-10/ESAT-6-specific CD8 T cell response in individuals with LTBI and patients with TB disease. The dotted line indicates the cut-off (3.3%) that distinguishes individuals with LTBI and patients with TB disease, with 92% sensitivity and 100% specificity. An ROC curve is shown indicating the sensitivity and specificity of the proportion of CFP-10/ESAT-6-specific CD8 T cells that are Bcl-2+CD57−CD95− in distinguishing individuals with LTBI and patients with TB disease. An area under the ROC curve (AUC) analysis was performed to further evaluate the performance of these particular phenotypic expression profiles in distinguishing individuals with LTBI and patients with TB disease. (PDF) [file pone.0094949.s002.pdf]

Figure S3

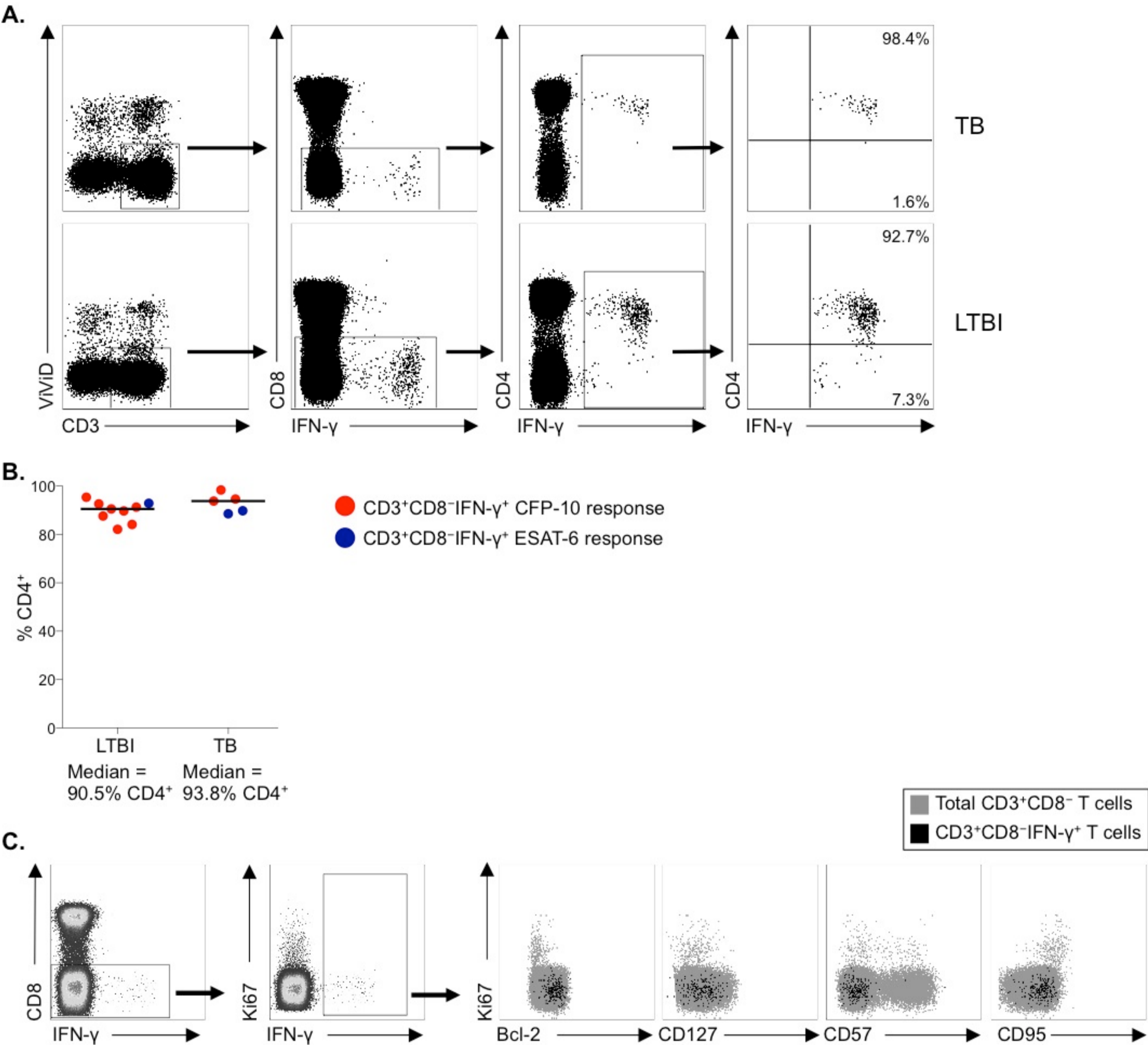

Supplement: Figure S3 — The majority of CFP-10 and ESAT-6-specific CD3+CD8−IFN-γ+ T cells are CD4+. PBMCs from individuals with LTBI and patients with TB disease were stimulated with CFP-10 and ESAT-6 peptide pools for 6 hours as described in the Materials and Methods section. Cells were stained with LIVE/DEAD Fixable Violet Dead Cell Stain (ViVid), anti-CD3 allophycocyanin-H7 (SK7), anti-IFN-γ Alexa Fluor 700 (B27), anti-CD8 PerCP-Cy5.5 (SK-1), all from BD Biosciences, and anti-CD4 QDot605 (S3.5) from Life Technologies. (A) Flow cytometry data representing the gating strategy for the analysis of CD4 expression on live CD3+CD8−IFN-γ+ T cells. Data are shown for PBMCs stimulated with CFP-10 peptide pool from a patient with TB disease (top row) and an individual with LTBI (bottom row). (B) Composite data indicating the percentage of CD3+CD8−IFN-γ+ T cells that are CD4+ in individuals with LTBI (n = 9) and patients with TB disease (n = 5). Each data point represents a single individual; colors indicate the antigen specificity of the response measured. (C) Flow cytometry data indicating the gating strategy used for phenotypic analysis of VIVIDl°CD3+CD8−IFN-γ+ cells. ESAT-6-specific IFN-γ+ cells from an individual with LTBI are shown as black dots overlayed on the total VIVIDl°CD3+CD8− population. (PDF) [file pone.0094949.s003.pdf]

Figure S4

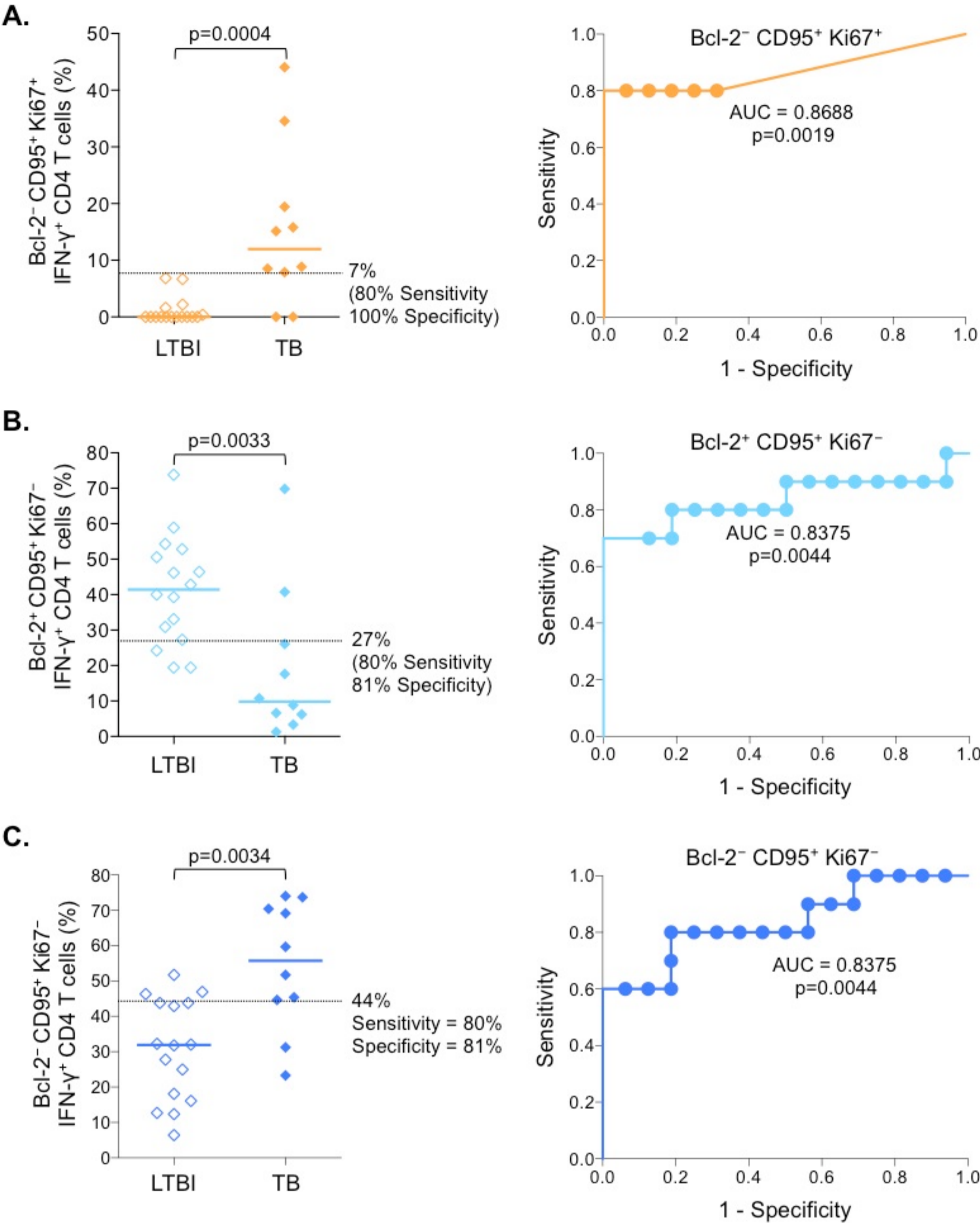

Supplement: Figure S4 — Predictive values of Bcl-2, CD95, and Ki67 expression by CFP-10/ESAT-6-specific CD4 T cells in distinguishing individuals with LTBI from TB disease patients. Co-expression patterns of Bcl-2, CD95, and Ki67 on CFP-10/ESAT-6-specific CD4 T cells were determined as described in Figure 3. (A) Comparison of the proportion of Bcl-2−CD95+Ki67+ cells contributing to the total CFP-10/ESAT-6-specific CD4 T cell response in individuals with LTBI and TB disease patients. The dotted line indicates the cut-off (7%) that distinguishes individuals with LTBI and patients with TB disease, with 80% sensitivity and 100% specificity. An ROC curve is shown indicating the sensitivity and specificity of the proportion of CFP-10/ESAT-6-specific CD4 T cells that are Bcl-2−CD95+Ki67+ in distinguishing individuals with LTBI and TB disease patients. (B) Comparison of the proportion of Bcl-2+CD95+Ki67− cells contributing to the total CFP-10/ESAT-6-specific CD4 T cell response in individuals with LTBI and TB disease patients. The dotted line indicates the cut-off (27%) that distinguishes individuals with LTBI from TB disease patients, with 80% sensitivity and 81% specificity. An ROC curve is shown indicating the sensitivity and specificity of the proportion of CFP-10/ESAT-6-specific CD4 T cells that are Bcl-2+CD95+Ki67− in distinguishing individuals with LTBI and TB disease patients. (C) Comparison of the proportion of Bcl-2−CD95+Ki67− cells contributing to the total CFP-10/ESAT-6-specific CD4 T cell response in individuals with LTBI and TB disease patients. The dotted line indicates the cut-off (44%) that distinguishes individuals with LTBI and patients with TB disease, with 80% sensitivity and 81% specificity. An ROC curve is shown indicating the sensitivity and specificity of the proportion of CFP-10/ESAT-6-specific CD4 T cells that are Bcl-2−CD95+Ki67− in distinguishing individuals with LTBI and TB disease patients. For panels A, B, and C, an area under the ROC curve (AUC) analysis was perfor [file pone.0094949.s004.pdf]
